# Increased risk of second malignancies after *in situ* breast carcinoma in a population-based registry

**DOI:** 10.1038/sj.bjc.6603231

**Published:** 2006-06-27

**Authors:** I Soerjomataram, W J Louwman, M J C van der Sangen, R M H Roumen, J W W Coebergh

**Affiliations:** 1 Department of Public Health, Erasmus MC, PO Box 2040, Rotterdam 3000 CA, The Netherlands; 2 Comprehensive Cancer Centre South, PO Box 231, Eindhoven 5600 AE, The Netherlands; 3 Department of Radiotherapy, Catharina Hospital, PO Box 1350, Eindhoven 5602 ZA, The Netherlands; 4 Department of Surgery, Maxima Medisch Centrum, PO Box 7777, Veldhoven 5500 MB, The Netherlands

**Keywords:** breast carcinoma *in situ*, population-based, risk, second cancer

## Abstract

Among 1276 primary breast carcinoma *in situ* (BCIS) patients diagnosed in 1972–2002 in the Southern Netherlands, 11% developed a second cancer. Breast carcinoma *in situ* patients exhibited a two-fold increased risk of second cancer (standardised incidence ratios (SIR): 2.1, 95% confidence interval (CI): 1.7–2.5). The risk was highest for a second breast cancer (SIR: 3.4, 95% CI: 2.6–4.3; AER: 66 patients per 10 000 per year) followed by skin cancer (SIR: 1.7, 95% CI: 1.1–2.6; AER: 17 patients per 10 000 per year). The increased risk of second breast cancer was similar for the ipsilateral (SIR: 1.9, 95% CI: 1.3–2.7) and contralateral (SIR: 2.0, 95% CI: 1.4–2.8) breast. Risk of second cancer was independent of age at diagnosis, type of initial therapy, histologic type of BCIS and period of diagnosis. Standardised incidence ratios of second cancer after BCIS (SIR: 2.3, 95% CI: 1.8–2.8) resembled that after invasive breast cancer (SIR: 2.2, 95% CI: 2.1–2.4). Surveillance should be directed towards second (ipsi- and contra-lateral) breast cancer.

Diagnosis of ductal breast carcinoma *in situ* has increased in the Netherlands, partly as a consequence of screening (Fracheboud *et al*, 2004), from 0.3 per 100 000 in 1975 to 13.4 per 100 000 in 1997 (Voogd *et al*, 2000). Women with previous breast cancer are known to carry a two-fold increased risk of second cancer in comparison to the general population (Rubino *et al*, 2003; Soerjomataram *et al*, 2005a). Studies assessing the risk of second cancer following the diagnosis of BCIS (breast carcinoma *in situ*) are however scarce or only focused on the risk of second breast cancer (Habel *et al*, 1997; Warnberg *et al*, 2000; Claus *et al*, 2003; Levi *et al*, 2005; Rawal *et al*, 2005).

An increased risk of 2.0–7.2 for breast cancer following the diagnosis of BCIS has been reported (Franceschi *et al*, 1998; Rawal *et al*, 2005), the probability that a breast cancer will develop in BCIS patients being 26% after 20 years of follow-up (Levi *et al*, 2005). This is as high as the risk of second breast cancer found for patients with malignant breast carcinoma (Chen *et al*, 2001). Excess risk of second breast cancer is not explained by treatment choice (i.e. radiotherapy) for BCIS (Claus *et al*, 2003), suggesting a shared aetiology (hereditary or lifestyle) for both first and second cancer. In addition to second breast cancer, other cancers were diagnosed in 17% of DCIS and 3.2% of lobular carcinoma *in situ* (LCIS) patients (Ward *et al*, 1992). However, no previous studies assessed the risks of different types of second cancer in BCIS patients.

The aim of the study is to assess the risk pattern for second cancer after diagnosis of BCIS and to compare it with that found for malignant breast carcinoma, thereby examining the impact of age, breast cancer screening policy at the time of primary BCIS diagnosis and treatment for various subtypes of BCIS.

## MATERIALS AND METHODS

### Data collection

Data were obtained from the population-based ECR (Eindhoven Cancer Registry) in the Southern Netherlands, covering 2.4 million inhabitants in 2004. The cancer registry is notified of newly diagnosed cases by the pathology departments in the region. In addition, lists of all hospitalised cancer patients were obtained. Active follow-up of vital status was conducted through the Central Bureau for Genealogy that receives data from municipal population registries. In the ECR, any new tumour, not classified as a recurrence or direct extension of a previously known tumour, is recorded as a new primary tumour. This registry also records incidence data on first basal cell carcinoma (BCC) of the skin. A detailed description of the data collection has been presented elsewhere (Soerjomataram *et al*, 2005a).

### Study population

We identified 1402 women older than 25 years diagnosed with *in situ* breast cancer (ICD-O behaviour code/2) from 1 January 1972 through 31 December 2002. Among patients eligible for the study, those with less than 1-year follow-up time (*n*=174) and those with unknown morphological code (*n*=5) were excluded. End of follow-up was 31 December 2003, date of death, date of last follow-up or date of second cancer diagnosis, whichever occurred first. Thus, 1223 women remained for analysis, 143 of whom (11.2%) developed a second cancer, 170 (13.3%) died and 2 (0.2%) were lost to follow-up. The maximum follow-up time was of 32 years.

### Statistical methods

We calculated standardised incidence ratios (SIRs) to measure the relative risk of developing second tumours by comparing the incidence of second cancer among patients with a diagnosis of BCIS to the incidence of similar cancer in the general population. We adjusted for age (in 5-year categories) and calendar year of BCIS diagnosis. The 95% confidence intervals (95% CI) were calculated using exact Poisson probability (Breslow and Day, 1987). We also calculated the absolute excess risk (AER) examining the excess incidence of second cancers per 10 000 patients in each year (van Leeuwen and Travis, 2005). Furthermore, the cumulative risk of developing second cancer, which is the proportion of patients alive at time *t* who can be expected to develop a second cancer, was calculated using the life table method (Cutler and Ederer, 1958).

The following categorisation of BCIS histological type was made; LCIS (ICD-O 8520/2) and DCIS including Paget's disease (ICD-O 8500/2, 8010/2, 8050/2, 8140/2, 8201/2, 8230/1, 8501/2, 8503/2, 8504/2, 8507/2, 8521/1, 8523/2, 8540/2) (Vereniging van Integrale Kankercentra, 2005). Year 1993 was considered the starting point of breast cancer screening, which was fully implemented in 1996 (Fracheboud *et al*, 1998, 2004). Calculation of risk for the ipsilateral and contralateral second breast cancer was performed using only patients with information on laterality of first BCIS and second breast cancer (excluded for this analysis *n*=52).

Standardised incidence ratios for selected cancers after malignant breast cancer were obtained from a previous study performed in ECR (Soerjomataram *et al*, 2005a) and compared with that of BCIS in the current study. In the earlier study, we estimated the risk of subsequent cancers in 9919 women diagnosed with malignant breast cancers in 1972–2000 followed until 2001. To allow comparison, we added second nonmelanotic skin cancer cases (BCC: 192 and squamous cell carcinoma: 42) for the analysis of second skin cancer. We used a similar method to calculate SIRs and 95% confidence intervals as explained before. A detailed description of this study has been described elsewhere (Soerjomataram *et al*, 2005a).

All statistical analyses were performed using SPSS 11.5 for Windows (Statistical Products and Service Solution, Inc., Chicago, IL, USA).

## RESULTS

The mean follow-up time for the cohort was 6.3 years. A large proportion of BCIS patients was older than 50 years and was diagnosed with DCIS (95%) in 1993–2002 (Table 1).

Table 2 shows the SIRs and AERs for second breast and other cancers. We found an increased risk of second breast cancer (SIR: 2.1, 95% CI: 1.7–2.5) and other non-breast cancers (SIR: 1.4, 95% CI: 1.1–1.9). An excess of 66 patients with second breast cancer for every 10 000 BCIS patients per year was observed. An increased risk of second breast cancer was found for both the ipsilateral (SIR: 1.9, 95% CI: 1.3–2.7) and contralateral breast (SIR: 2.0, 95% CI: 1.4–2.8). Almost a two-fold elevated risk of skin cancer (SIR: 1.7, 95% CI: 1.1–2.5) was found.

A three- to four-fold increased risk of second breast cancer was found during the first 10 years of follow-up (Table 3), which was relatively higher than the SIR for the last follow-up period (≥10 years). As for the risk of second non-breast cancer, we observed similar SIRs across all follow-up periods.

**Figure 1:**
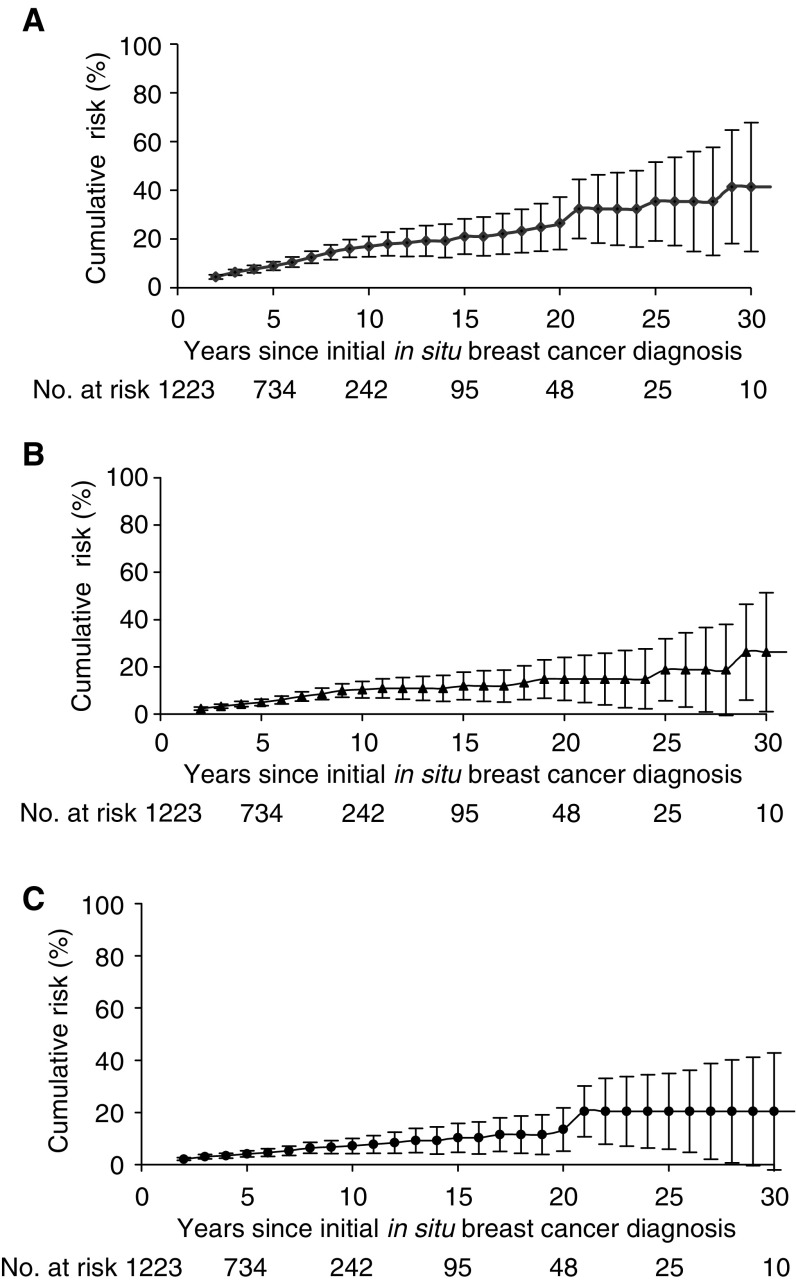
Cumulative risk of second cancer after the diagnosis of carcinoma *in situ* of the breast: (**A**) all sites, (**B**) breast cancer, (**C**) other sites excluding breast. No. at risk represented patients still at risk at the beginning of each period.

Increased risks of second breast or other cancers were not influenced by age at BCIS diagnosis, type of initial therapy, histological type of BCIS and time of BCIS diagnosis (Table 4). Ipsi- and contra-lateral invasive breast cancer risks were slightly higher for BCIS patients who received radiotherapy (SIR: 2.1, 95% CI: 1.0–4.0 and SIR: 2.4, 95% CI: 1.2–4.3, respectively), compared to patients who did not receive radiotherapy (SIR: 1.7, 95% CI: 1.1–2.8 and SIR: 1.8, 95% CI: 1.1–2.8). The cumulative 10-year risk of developing any second cancer was 17% (±5%), whereas the 15-year corresponding risk was 21% (±8%) (Figure 1).

**Figure 2:**
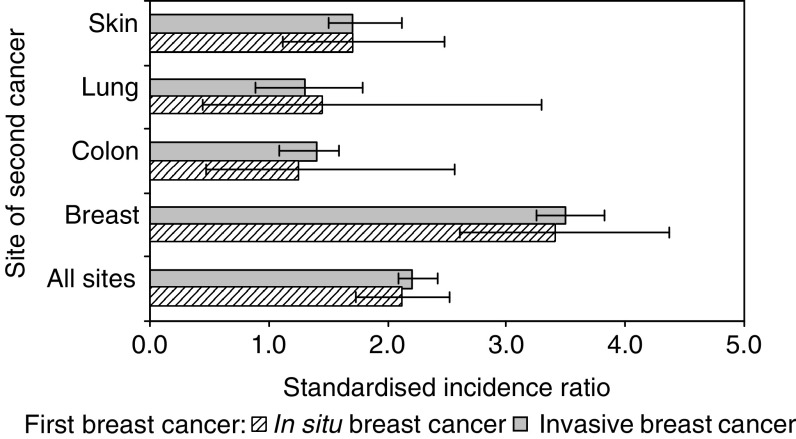
Standardised incidence ratio for second cancer among women diagnosed with breast carcinoma *in situ* and with invasive breast carcinoma (Soerjomataram *et al*, 2005a).

Figure 2 compares the SIRs for second cancer after BCIS with those after invasive breast cancer for selected malignancies. The SIRs for second cancer of the lung, colon, skin and breast after BCIS were similar to those after invasive breast cancer. The risk pattern of second cancer at all sites after BCIS (SIR: 2.3, 95% CI: 1.8–2.8) was similar to that of second cancer after invasive breast cancer (SIR: 2.2, 95% CI: 2.1–2.4).

## DISCUSSION

Women previously diagnosed with *in situ* breast carcinoma had an increased risk of second cancer, in particular second breast and skin cancer. An excess of 90 second cancers per 10 000 BCIS patients was found. Similar to previous studies (Franceschi *et al*, 1998), we observed a 21% increased risk for a second cancer after 15 years of survival.

Some limitations of our study should be considered. Firstly, as most women were diagnosed after 1993, the majority had less than 10 years of follow-up. Furthermore, the absolute numbers of our study is relatively small. Thus, we may not have estimated correctly the long-term risk of less common cancers such as ovarian cancer, which exhibits an increased risk among long-term survivors of invasive breast cancer (Soerjomataram *et al*, 2005b). Secondly, increased medical surveillance of women with a diagnosis of BCIS may have increased detection of second cancers (van Leeuwen and Travis, 2005). In our cohort, 60% (30 patients) were diagnosed with second cancer within the first year after BCIS diagnosis. Therefore, we excluded patients with less than 1-year of follow-up and those with a second carcinoma *in situ*. Thirdly, AER in this article should be interpreted with caution because BCIS accounts for only approximately 13% of all breast cancer diagnoses (Fracheboud *et al*, 2004). Thus, given the same AER, the absolute number of second cancers after BCIS will be considerably smaller than that after invasive breast cancer at the population level. Lastly, no individual data were available on risk factors for cancer (Soerjomataram *et al*, 2005b). Hence, the contribution of these factors to the risk of second cancer could not be assessed.

### Risk pattern

After the diagnosis of BCIS, there was an increased risk of second breast and skin cancer. The question is whether second malignancies share a common aetiology with the first cancer or whether they are associated with treatment for the first cancer (van Leeuwen and Travis, 2005). It is likely that factors including reproductive characteristics, lifestyle and genetic predisposition such as BRCA2 play a more important role in the excess risk of both second breast and skin cancer after BCIS (Arver *et al*, 2000; Goggins *et al*, 2004). We did not find an increased risk of second ovarian cancer among BCIS patients as in patients with malignant breast cancer. However, most patients in this study had less than 10 years of follow-up and the risk of ovarian cancer after breast cancer was highest after more than 15 years of follow-up (Soerjomataram *et al*, 2005b).

### Determinants

#### Age

Age at the time of BCIS diagnosis did not seem to influence the risk for second cancer, although we observed a slightly higher risk of second breast cancer among women diagnosed with BCIS before age 50. A higher risk of second breast cancer has been found among *in situ* and malignant breast cancer patients diagnosed before the age of 50 years (Rawal *et al*, 2005; Soerjomataram *et al*, 2005b). This is partly due to genetic predisposition, which usually becomes manifest at a relatively young age.

#### Treatment

The risk for second (ipsi- and contra-lateral) breast and other cancers was slightly higher among BCIS patients who received radiotherapy. Radiation after breast-conserving treatment reduces recurrences in the ipsilateral breast (Fisher *et al*, 1993; Julien *et al*, 2000), but its effect on the risk of new (ipsi- or contra-lateral) breast cancer is less conclusive (Warnberg *et al*, 2001; Claus *et al*, 2003). We found only a slightly increased risk of second breast cancer after radiation that was not significantly different from that of patients without radiotherapy. Thus, the benefit of radiation after surgery for the overall survival of DCIS patients seems to outweigh the increased risk of second breast cancer (Fisher *et al*, 1993).

#### Screening

The risk of second cancer after BCIS remained elevated and of a similar magnitude after implementation of the national screening policy in the Netherlands. In Sweden, the risk of second breast cancer increased at the beginning of the screening period and only decreased after long implementation of national screening (Rawal *et al*, 2005). Thus, in the coming decades, we might observe a decrease in the risk of second cancer after BCIS.

### Comparison with invasive breast cancer cohort

The pattern of second cancer after BCIS seems to be similar to that after malignant breast cancer. Cancers of the colorectum, ovarium, lung and skin were some of the most common cancers in women previously diagnosed with an invasive breast cancer (Soerjomataram *et al*, 2005a). In the USA, colorectal, cervical and endometrial cancer were reported as the most prevalent cancers among BCIS patients (Ward *et al*, 1992). Thus, we could probably expect an increased incidence of second cancers resembling that of malignant breast cancer within a larger study population and a longer follow-up of BCIS cases.

In conclusion, we found increased relative and absolute risks of second cancer after BCIS diagnosis, similar to that after invasive breast cancer. Monitoring for these breast cancers should therefore be conducted in both the ipsilateral and the contralateral breast.

## Figures and Tables

**Table 1:** Characteristics at diagnosis of BCIS (breast carcinoma *in situ*)^a^

	**Subsequent cancer**		
	**No. (%)**	**Yes (%)**	**Total (%)**
*Age at BCIS diagnosis*			
≤49 years	288 (26)	30 (27)	318 (26)
≥50 years	822 (74)	83 (73)	905 (74)
			
*Initial treatment*			
No radiotherapy	765 (69)	68 (60)	833 (68)
With radiotherapy	345 (31)	45 (40)	390 (32)
			
*Follow-up*			
1–4 years	549 (49)	64 (57)	613 (50)
5–9 years	396 (36)	34 (30)	430 (35)
≥10 years	165 (15)	15 (13)	180 (15)
			
*Subtype of initial cancer*			
DCIS^b^	1052 (95)	105 (93)	1157 (95)
LCIS^c^	58 (5)	8 (7)	66 (5)
			
*Time of diagnosis* d			
1972–1992	165 (15)	41 (36)	206 (17)
1993–2002	945 (85)	72 (64)	1017 (83)
			
Total	1110	113	1223 (100)

a Mean age at BCIS diagnosis=57.1 years; Mean follow-up time=6.3 years

b DCIS: ductal carcinoma *in situ*.

c LCIS: lobular carcinoma *in situ*.

d Breast cancer screening in southern Netherlands began to have impact in 1993 (Fracheboud *et al*, 2004).

**Table 2:** Standardised incidence ratio (SIR) and absolute excess risk (AER) for all second cancers diagnosed in 1972–2003 following BCIS (breast carcinoma *in situ*) in southern Netherlands

	**Relative and absolute risks^a^**				
**Site of second cancer**	**Observed**	**Expected**	**SIR**	**95% CI**	**AER**
All sites	113	54.4	2.1^b^	1.7–2.5	90
All sites excluding breast^c^	52	36.2	1.4^b^	1.1–1.9	24
					
*Digestive tract* d	11	10.4	1.1	0.5–1.9	1
Stomach	3	1.6	1.8	0.4–5.3	2
Colon	6	5.2	1.2	0.4–2.5	1
					
Lung	5	3.5	1.4	0.5–3.3	2
*Skin* e	27	15.8	1.7^b^	1.1–2.5	17
Melanoma	4	1.4	3.0	0.8–7.6	4
Basal cell carcinoma	22	12.8	1.7^b^	1.1–2.6	14
					
*Breast*	61	18.1	3.4^b^	2.6–4.3	66
Ipsilateral^f^	29	15.5	1.9^b^	1.3–2.7	24
Contralateral^f^	31	15.5	2.0^b^	1.4–2.8	28
					
*Urogenital tract* g	4	7.1	0.6	0.2–1.4	−5
Ovary	2	2.5	0.8	0.1–2.8	−1
					
Lymphoma and multiple myeloma	2	2.5	0.8	0.1–2.9	−1

a Excluding patients with less than 1-year follow-up.

b 95% confidence interval excludes 1.

c Three observed are primary cancers of unknown origin.

d Also includes pancreas [1] and rectum [1].

e Also includes squamous cell carcinoma of the skin [1]

f Only includes patients with known laterality of BCIS and second breast cancer.

g Also includes corpus uteri [1] and bladder [1].

**Table 3:** Standardised incidence ratio (SIR) and absolute excess risk (AER) for second breast cancer and second other cancers after BCIS (breast carcinoma *in situ*), according to follow-up time

		**Second breast cancer**				**Other second cancers**			
**Period of follow-up**	**PYR^a^**	**Obs^b^**	**Exp^c^**	**SIR**	**AER**	**Obs^b^**	**Exp^c^**	**SIR**	**AER**
1–4 years	3596	33	9.7	3.4^d^	65	31	18.9	1.6^d^	34
5–9 years	1815	22	5.0	4.4^d^	94	12	10.1	1.2	11
≥10 years	1127	6	3.3	1.8	24	9	7.4	1.2	14

a PYR: person-years.

b Obs: observed numbers of second primary cancers.

c Exp: expected numbers of second primary cancers.

d 95% confidence interval excludes 1.

**Table 4:** Standardised incidence ratio (SIR) and absolute excess risk (AER) for all second cancers diagnosed 1972–2003 following BCIS (breast carcinoma *in situ*) in southern Netherlands, according to women's characteristics at the time of BCIS diagnosis

		**Second breast cancer**				**Second other cancers**			
**Characteristic**	**PYR^a^**	**Obs^b^**	**Exp^c^**	**SIR**	**AER**	**Obs^b^**	**Exp^c^**	**SIR**	**AER**
*Age at diagnosis*									
≤49 years	2334	20	4.9	4.0^d^	65	10	6.6	1.5	15
≥50 years	4204	41	13.1	3.1^d^	66	42	29.8	1.4^d^	29
									
*Treatment*									
No Radiotherapy	4474	39	12.4	3.1^d^	59	29	25.2	1.1	8
With Radiotherapy	2064	22	5.6	3.9^d^	79	23	11.1	2.1^e^	57
									
*Subtype of initial cancer*									
DCIS	6106	58	16.8	3.4^d^	67	47	34.3	1.4^d^	21
LCIS	432	3	1.2	2.5	42	5	2.1	2.4	67
									
*Time of diagnosis* e									
1972–1992	2708	24	7.0	3.4^d^	63	17	14.4	1.2	9
1993–2002	3830	37	11.0	3.4^d^	68	35	21.9	1.6^d^	34

a PYR: person-years.

b Obs: observed numbers of second primary cancers.

c Exp: expected numbers of second primary cancers.

d 95% confidence interval excludes 1.

e Breast cancer screening in southern Netherlands began to have impact in 1993 (Fracheboud *et al*, 2004).
